# Deep Learning-Enhanced Diagnosis of Sow Pregnancy Through Low-Frequency Ultrasound Imaging

**DOI:** 10.3390/ani15030318

**Published:** 2025-01-23

**Authors:** Tae-kyeong Kim, Yo-Han Choi, Jun-Seon Hong, Hyun-Ju Park, Yong-Min Kim, Jo-Eun Kim, Ji-Hwan Lee, Soo-Jin Sa, Yong-Dae Jeong, Jin-Soo Kim, Hyun-chong Cho

**Affiliations:** 1 Department of Data Science, Kangwon National University, Chuncheon 24341, Republic of Korea; rlaxorud0219@kangwon.ac.kr; 2 Swine Science Division, National Institute of Animal Science, Rural Development Administration, Cheonan 31000, Republic of Korea; cyh6150@korea.kr (Y.-H.C.); gospel0342@korea.kr (J.-S.H.); 77qk77@naver.com (H.-J.P.); silveraz@korea.kr (Y.-M.K.); kjektw@korea.kr (J.-E.K.); junenet123@naver.com (J.-H.L.); soojinsa@korea.kr (S.-J.S.); yongdaejeong@korea.kr (Y.-D.J.); 3 College of Animal Life Sciences, Kangwon National University, Chuncheon 24341, Republic of Korea; 4 Department of Electronics Engineering, Interdisciplinary Graduate Program for BIT Medical Convergence, and Department of Data Science, Kangwon National University, Chuncheon 24341, Republic of Korea

**Keywords:** deep learning, low-frequency ultrasound, pregnant diagnosis

## Abstract

The escalating demand for pork highlights the importance of swift and accurate pregnancy diagnosis in sows, a crucial factor in farm profitability. The prevalent use of low-frequency ultrasound devices in this context poses a challenge owing to the suboptimal resolution of the resultant images. This study introduces an innovative approach for sow pregnancy diagnosis using deep learning techniques to analyze low-frequency ultrasound images. Our methodology encompasses the development and comparative analysis of three distinct classification models: ViT-H, ConvNeXt-xlarge, and Xception. These models aim to improve diagnostic accuracy. AutoAugment was used to augment the data to expand the training dataset, thereby enhancing the robustness of the models under varied conditions. Results indicate a notable improvement in diagnostic performance, with the implementation of AutoAugment leading to significant achievements in the models, reflected by AUC values of 0.865, 0.856, and 0.866. These outcomes affirm the viability of deep learning in the effective management of sow pregnancies in livestock farms and suggest potential applications in broader animal husbandry contexts. This research marks a significant contribution to the evolution of agricultural technologies, presenting a scalable and efficacious solution for sow pregnancy diagnosis.

## Simple Summary

Early and accurate pregnancy detection in sows is critical for improving farm management. However, using ultrasound technology, especially low-frequency devices, often produces images with poor quality, making it difficult to diagnose pregnancy accurately. This study introduces a new approach that applies deep learning to analyze ultrasound images and enhance the accuracy of pregnancy detection. By testing three different models, this method achieves significantly better results than traditional techniques. This new approach could help farmers detect pregnancy earlier and more reliably, improving the health and management of livestock while increasing farm profitability.

## 1. Introduction

**Figure 1:**
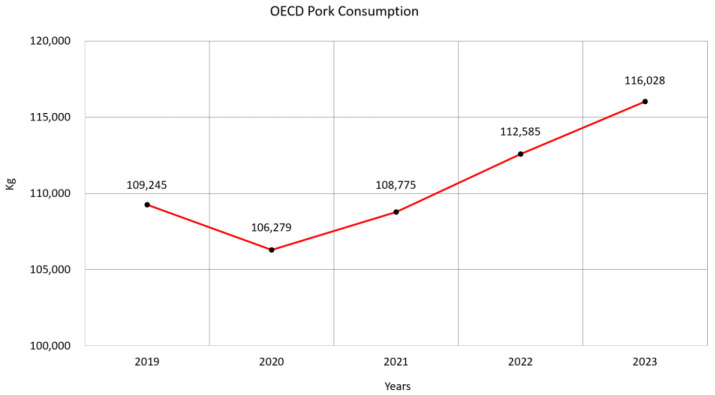
Quantification of pork consumption in OECD countries.

When analyzing global meat consumption, pork consumption exhibits a certain degree of variability due to African Swine Fever (ASF) but remains a significant factor. This trend is reflected in the pork consumption data of Organization for Economic Cooperation and Development (OECD) countries, as shown in Figure 1 [1].

In this context, it is essential to produce pork and ensure efficiency and stability throughout the production process. As the demand for pork increases sharply, optimizing productivity has become crucial for the operation of farms and livestock businesses and the economic efficiency of the entire supply chain. In particular, as evidenced in various studies, effective management during reproductive and gestational stages in sows is directly linked to improved productivity. Nutritional and stress management during early and mid-pregnancy are particularly critical. However, in group housing systems, regulating feed intake for individual sows is challenging, which can lead to reproductive failures [2]. The method of feed allocation for individual sows also plays a significant role. Supplementation with n-3 fatty acids during early pregnancy has been shown to reduce embryonic mortality, shorten the weaning to estrus interval, improve conception rates, and enhance piglet birth weights [3]. Additionally, recent genetic improvements have increased litter sizes and birth weights, leading to higher amino acid requirements for sows [4]. Specifically, lysine requirements have been found to increase by approximately 31% during late pregnancy. Furthermore, phase-feeding programs and targeted supplements have significant potential to improve reproductive performance. Supplements containing lysine, arginine, and methionine can greatly contribute to fetal growth. Feeding programs that meet these nutritional demands can mitigate reproductive failures and improve piglet weight and health outcomes [5].

Therefore, an efficient and scientific approach to managing sow pregnancy and reproduction has emerged as a critical factor from an economic perspective, and it is becoming an essential management element closely tied to changes in consumer demand [6]. Additionally, early pregnancy detection in sows enables timely nutritional management, which can significantly contribute to improved productivity.

Various methods have been validated for accurate sow pregnancy diagnosis. Notably, the measurement of estrone sulfate and estrogen levels in blood, urine, and feces has been recognized as a reliable early diagnosis technique [7]. This method hinges on detecting specific hormonal thresholds: estrone sulfate levels exceeding 2.79 ng/mL in blood, estrogen levels surpassing 19.7 ng/g in feces, and estrogen concentrations exceeding 65.6 ng/mL in urine. A study involving 289 sows, which entailed collecting 25 μL blood samples from the tail vein for enzyme-linked immunosorbent assay (ELISA), reported an accuracy rate of 91.7%. Additionally, urine samples (1 mL) collected from 100 sows using a cellulose sponge and 2 g fecal samples from 290 sows analyzed through radioimmunoassay (RIA) demonstrated accuracy rates of 98% and 93.5%, respectively.

Further research on hormonal markers of progesterone and estrone sulfate in 51 sows’ blood samples reinforces these findings. In this study, pregnancy detection was possible at progesterone levels above 5 ng/mL and estrone sulfate levels above 0.5 ng/mL [8], with blood samples drawn 19–23 days and 26–30 days post-estrus, analyzed using RIA. The results were notable, with 98% accuracy for estrus detection and 92% accuracy for both estrone sulfate and progesterone measurements.

Hormonal marker-based approaches, while effective, often involve invasive procedures such as blood collection, raising concerns about stress and potential injury in sows. Despite the high accuracy of these invasive methods, their potential stress induction and associated infection or injury risks in sows are concerning. For instance, invasive procedures during pig management can cause infections and injuries, leading to pain and stress [9]. Pain can also induce behavioral, autonomic, and neuroendocrine changes, potentially deteriorating the physiological and emotional health of the animals [10]. These findings highlight the necessity of exploring less invasive alternatives for improved animal welfare. This study uses ultrasound technology and deep learning methods to address these concerns and provide a non-invasive, efficient, and accurate diagnostic approach for sow pregnancy. One study has explored non-invasive diagnostic techniques using metabolomics, such as biomarkers for early pregnancy detection [11]. This research involved six nonpregnant and six pregnant sows, identifying Hyodeoxycholic acid and 2′-deoxyguanosine in saliva as promising diagnostic indicators with high AUC values. While this approach has significant potential as a non-invasive diagnostic tool that considers animal welfare, it is limited by a small sample size and the need for further validation in practical farming environments.

Another study suggested a low-cost method for diagnosing pregnancy in pigs using a seed germination inhibition test [12]. This study, conducted on 315 sows, found that the mean seed germination inhibition rate in the pregnant group was significantly higher at 75.66% ± 3.48 compared to the nonpregnant group (28.70% ± 2.96) and the control group (19.48% ± 2.69) (*p* < 0.05).

This study emphasizes a simple and feasible approach for resource-limited regions but acknowledges certain limitations. The seed germination inhibition rate may vary depending on seed type, urine concentration, and environmental conditions. Additionally, the hormone concentration in urine can fluctuate due to physiological factors such as health status, hydration levels, and stress. Considering these limitations, ultrasound technology has gained attention as a reliable pregnancy diagnosis method if it can provide non-invasive and generalized images.

A study utilizing Doppler Echo+ and other ultrasound devices revealed varying levels of sensitivity, specificity, and efficiency, highlighting the nuanced performance of ultrasound-based diagnostics in early pregnancy detection. For instance, Doppler Echo+ used on 107 sows achieved 85% sensitivity, 32% specificity, and 73% overall efficiency. Moreover, ultrasonic evaluations on 142 sows, during early (17 to 24 days) and later (38 to 45 days) gestation periods, highlighted the potential of real-time ultrasound in achieving over 90% sensitivity, 45% specificity, and 70% efficiency after 21 days of gestation [13]. Another study proposed pregnancy diagnosis using real-time B-mode ultrasound. This study was conducted on 30 sows and demonstrated that a professional operator could diagnose pregnancy 20 days after insemination. However, this method has limitations, including high costs and training personnel requirements [14].

Early pregnancy diagnosis is vital in farm settings because it plays a key role in minimizing miscarriages and ensuring timely nutritional support for pregnant sows [15]. This early detection is instrumental in enhancing farm profitability by reducing extended non-gestation periods, which could disrupt farm operations [16]. Furthermore, physiological studies reveal significant changes in uterine echogenicity during early pregnancy that could serve as reliable markers for detection [17]. Research utilizing B-mode ultrasound and gray-scale analysis has demonstrated that changes in uterine tissue echogenicity are observable as early as 15 days post-breeding, highlighting the potential for early and accurate pregnancy diagnosis. These findings emphasize the biological feasibility of early detection and its integration into farm management practices.

Pig gestation evaluation typically involves agricultural personnel manually inspecting ultrasonographic equipment. However, this method is susceptible to subjective interpretation and is influenced by the expertise and experience of the observer, which can lead to potential misclassifications, such as incorrectly identifying pregnant sows as nonpregnant [18]. Moreover, traditional methods often focus on identifying the gestational sac, limiting the feasibility of early pregnancy detection. These issues are not exclusive to agriculture but are also prevalent in various medical applications. To overcome these limitations, deep learning methodologies have been increasingly integrated across sectors to analyze ultrasonic scans, as evidenced in previous studies [19].

Recent studies highlight the advantages of using a 5MHz transducer, which offers improved imaging and higher diagnostic accuracy for pregnancy detection in sows [20]. This emphasizes the importance of equipment optimization for achieving reliable results. However, using low-frequency ultrasound devices in practical farm environments poses challenges, primarily due to reduced image clarity, which can impact clinical management effectiveness by up to 70% [21]. In summary, existing methods for sow pregnancy diagnosis exhibit varying accuracy, efficiency, and practicality, yet the need for a reliable, non-invasive, and cost-effective approach remains. This study addresses this gap by developing an ultrasound-based diagnostic method enhanced with deep learning to improve accuracy and practicality in real farm environments. Specifically, the study seeks to compare the performance of Transformer-based models with convolutional neural network (CNN) models and leverage data augmentation techniques to enhance pregnancy detection accuracy significantly. These advancements are expected to provide valuable insights for farm management, aligning with the growing global demand for pork.

## 2. Materials and Methods

### 2.1. Dataset

The dataset is a crucial element of this study and was approved by the Institutional Animal Care and Use Committee (IACUC) of the Rural Development Administration, under the approval number NIAS-2021-538. This study was conducted in compliance with ethical standards, ensuring the animals’ welfare. Data were collected from a commercial pig farm in Haman-gun, Gyeongsangnam-do, Republic of Korea. This dataset was meticulously collected by experts using low-frequency ultrasound imaging technology and was validated through cross-validation. Specifically, the SM-T530 tablet (Samsung, Seoul, Republic of Korea) was integrated with a 3.5 MHz transducer and an SV-1 wireless scanner (Clarius Mobile Health, Vancouver, BC, Canada). This device facilitates an imaging depth between 100 mm and 180 mm, making it suitable for detecting pregnancy in sows. Additionally, to ensure consistency and reproducibility in evaluation, all ultrasound images were acquired under the same parameter conditions and collected in video format. The dataset encompasses data from 551 sows, stratified by pregnancy status and gestational age into four groups: non-pregnant sows (*n* = 141) and sows at various stages of pregnancy: 15–17 days (*n* = 79), 18–21 days (*n* = 202), and 22–25 days (*n* = 129). Specialists performed all ultrasound scans following standardized protocols, ensuring consistent imaging quality across all samples. The inclusion criteria for sows were based on their pregnancy status and gestational age, verified through breeding records and ultrasonographic observations. While demographic factors such as breed and age were not explicitly controlled, the sows were sourced from a single commercial pig farm to ensure consistency in farming practices.

To ensure compatibility with the requirements of deep learning models trained on image-based data, videos were converted into images at 5 FPS intervals. This frame interval was chosen to enhance computational efficiency and prevent redundant images from being included in the training dataset, thereby maintaining a balanced representation. During the conversion process, the BMP format was used to preserve the original resolution of 1120 × 624 and to prevent data loss during preprocessing. Each sow generated 2 to 4 videos, each recorded at 10 s in length and a frame rate of 10 FPS.

To maintain the natural distribution of the data, only uterine images deemed inaccurate were removed through expert review, while all other data were retained. As a result, the curated final dataset consisted of 5792 images of non-pregnant sows, 2920 images from sows at 15 to 17 days of pregnancy, 8742 images at 18 to 21 days, and 6424 images at 22 to 25 days. This meticulous curation ensured that the model training process included only relevant and high-quality images. Additionally, identifying information such as IDs and dates was obscured to preserve anonymity and data integrity during the deep learning process.

The data were randomly divided into training and testing datasets following an 80:20 ratio. Careful measures were taken to ensure no overlapping sow IDs between these sets, avoiding potential model overfitting. This random division approach maintained the objectivity and reliability of the results by ensuring independence between the training and testing phases. The dataset was segmented based on different pregnancy stages, forming three distinct comparison groups: [nonpregnant sows/15–17 days pregnant sows], [nonpregnant sows/18–21 days pregnant sows], and [nonpregnant sows/22–25 days pregnant sows]. Nonpregnant sows were defined as individuals who underwent breeding but did not conceive. Due to challenges in collecting sufficient data for this group, images from the ‘nonpregnant’ sows were redistributed among the three comparison groups based on temporal proximity to ensure a balanced analysis. Table 1 in the study details the categorization of the dataset, providing a comprehensive and transparent overview of the data distribution, which underpins the rigorous training of deep learning models for this study.

### 2.2. Deep-Learning-Based Pregnant Sows Classification

In deep-learning-based image analysis, the methods can be broadly categorized into classification, object detection, and segmentation [22]. Classification tasks focus on identifying the category or class of a single object within an image, often serving as the foundation for more complex image analysis methods. Object detection, conversely, identifies the classes of multiple objects and determines their precise locations within the image by providing bounding boxes. Segmentation takes this a step further by delineating the exact boundaries of the objects identified through object detection down to the pixel level, enabling more detailed and granular analysis. In this study, we employ classification techniques to analyze low-frequency ultrasound images of sows segmented based on gestational age. The method effectively distinguishes between pregnant and nonpregnant states by utilizing deep learning models for classification, offering a non-invasive and efficient diagnostic approach. This approach highlights the potential of deep learning to enhance the accuracy and reliability of reproductive management in livestock.

**Figure 2:**
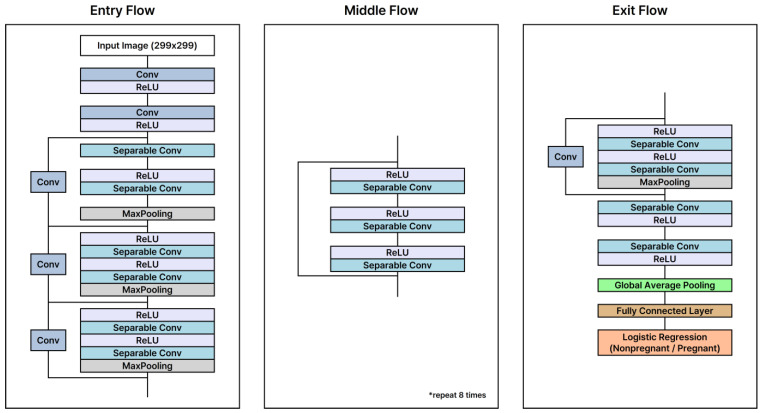
Architectural framework of the Xception model.

**Figure 3:**
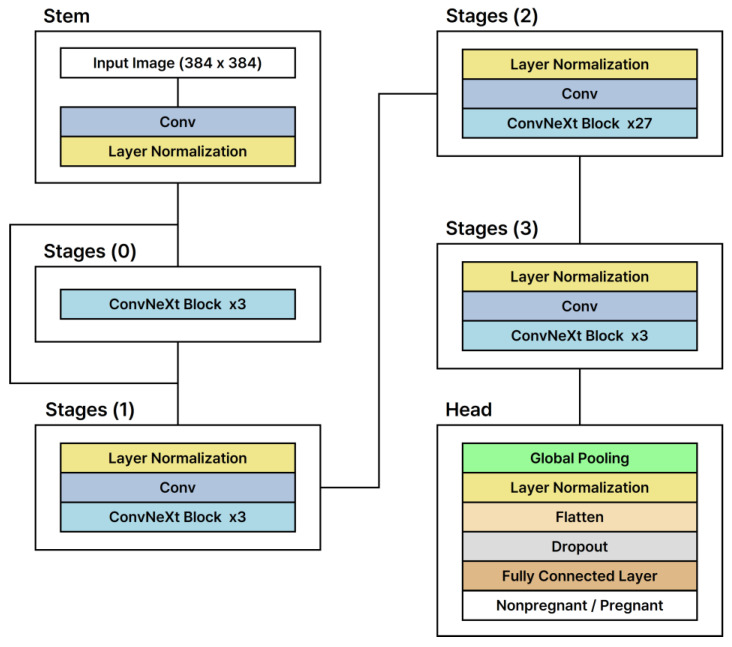
Architectural framework of the ConvNeXt-xlarge model.

**Figure 4:**
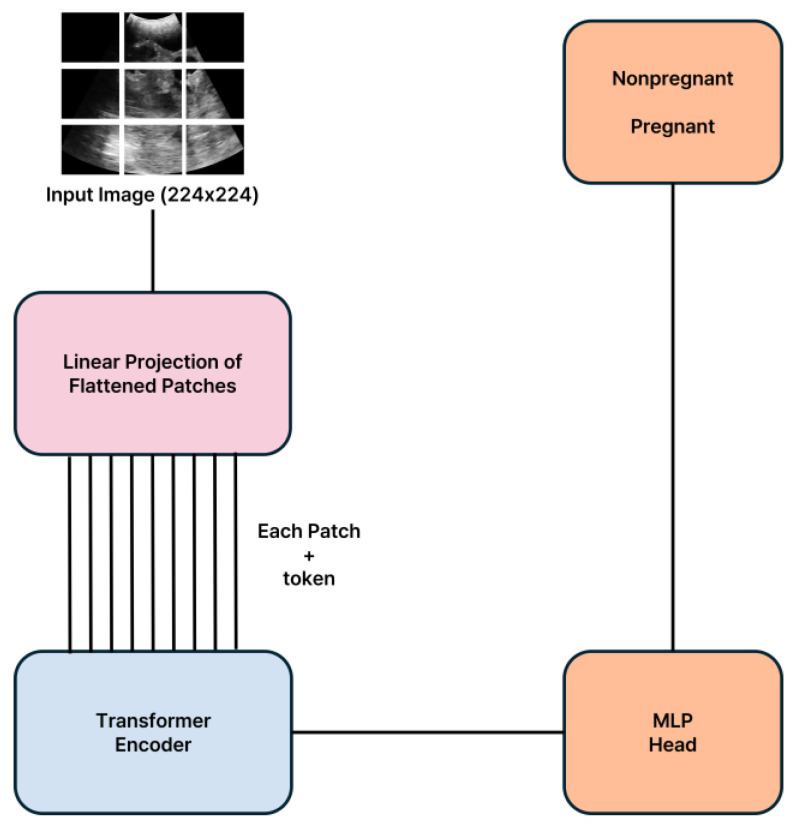
Architectural framework of the ViT-H model.

Our approach compares the efficacy of two convolutional neural network (CNN)-based models, ConvNeXt-xlarge and Xception, with a transformer-based model, ViT-H, to determine the most effective framework for analyzing low-frequency ultrasound images in pregnancy diagnosis. The ConvNeXt-xlarge model enhances conventional CNN architectures by incorporating elements from transformer models, enabling deeper and more extensive network capabilities [23]. This hybrid approach improves generalization while preserving the advantages of CNN-based feature extraction. The Xception model maximizes the potential of the Inception module by employing Depthwise Separable Convolutions, which balance efficiency and performance, making it particularly suitable for relatively small datasets [24]. In contrast, the ViT-H model, which is derived from the Transformer architecture originally developed for natural language processing, adapts it for image recognition. It divides an image into multiple patches as input for the Transformer, demonstrating efficacy in complex image recognition tasks with its deep network structure and extensive parameter set. This mechanism facilitates the recognition of global contexts within images, demonstrating efficacy in complex image recognition tasks with its deep network structure and extensive parameter set. The structures of these models are depicted in Figure 2, Figure 3 and Figure 4. All models were trained using early stopping, which stops training when the validation loss converges to avoid overfitting the training data. Performance metrics show that both CNN-based and Transformer-based models generalize well, and Transformer-based models show significant strength in capturing global image patterns.

The selection of these models reflects a strategic evaluation of their potential applications in real-world farm settings. The lightweight Xception model is evaluated for its practicality in environments with limited computational resources, while the computationally heavier ConvNeXt-xlarge and ViT-H models provide benchmarks for high-performance diagnostics. This comprehensive assessment aims to bridge the gap between advanced deep learning technologies and their applicability in practical swine reproductive management, ensuring that the proposed methodologies are innovative and implementable.

**Figure 5:**
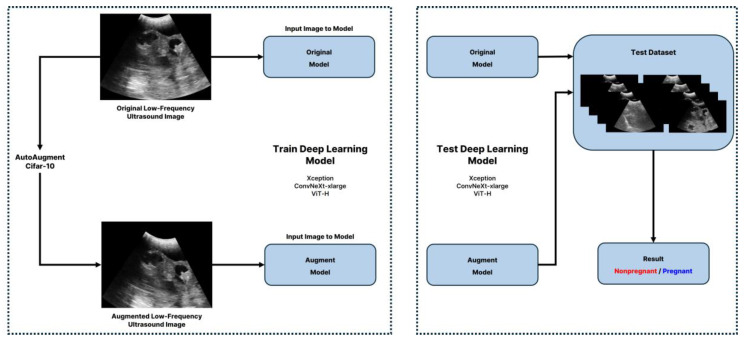
Diagrammatic representation of the proposed methodology’s workflow.

When a dataset contains a significant amount of noise, missing values, or is small, issues may occur that reduce the accuracy of machine learning models [25]. Additionally, in the case of small datasets, generalization errors tend to increase, posing further challenges [26]. While machine learning and deep learning models typically perform excellently on large datasets, environmental constraints can make data collection difficult. In such scenarios, data augmentation is a practical approach to enhance model performance by expanding the diversity of small datasets using existing data. This technique compensates for limited data availability and helps improve models’ robustness and generalization capability [27]. Based on this research, this study utilized Google’s AutoAugment method [28]. AutoAugment assesses various augmentation strategies to identify the most effective combinations for image datasets through rigorous testing on datasets such as Cifar-10, ImageNet, and SVHN [29,30,31]. It comprises 16 distinct augmentation techniques, including [CutOut], [Sample Pairing], [Rotate], [Shear X/Y], [Translate X/Y], [Auto Contrast], [Invert], [Equalize], [Solarize], [Posterize], [Contrast], [Brightness], [Sharpness], and [Color]. This study explicitly applies policies learned from the Cifar-10 dataset, featuring 32 × 32-pixel images across 10 classes. While AutoAugment was originally designed for color image datasets, its transformations are also effective for grayscale images, including ultrasound data where spatial and structural features are critical [32]. Given its proven efficacy in limited-class contexts, the Cifar-10 policy is apt for this study’s binary classification challenge. The research methodology, as illustrated in Figure 5, was designed based on the following to ensure robustness and practical applicability:

1. Standardized data preparation: The integrity of the data was maintained through a consistent process of converting ultrasound videos to lossless images in BMP format. This ensured data consistency in both the original and augmented datasets.

2. Data augmentation: The standardized data were augmented 25 times through Autoaugment.

3. Balanced model evaluation: The original and augmented datasets were trained under the same conditions using the same pre-trained models (ConvNeXt-xlarge, Xception, ViT-H). This was designed to ensure that the evaluation of the data augmentation effect was unbiased and directly comparable.

**Figure 6:**
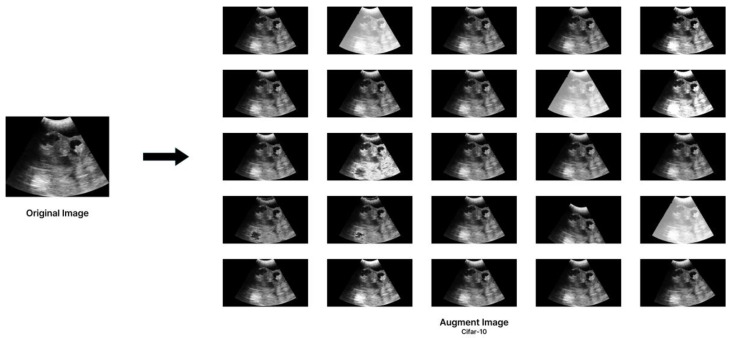
Illustrative comparison between the original and augmented data samples.

Computational experiments were conducted on a system equipped with an Intel(R) Core(TM) i9-10920X CPU, NVIDIA GeForce RTX 3090, and 64GB of Memory, utilizing Python 3.11.7, Pytorch 2.1.2, and Cuda 12.1. Each model was trained over 40 epochs with a batch size of 8, using the Adam optimizer at a learning rate of 0.0001 [33]. All models used in this study were pre-trained, ensuring consistency in the initial state across experiments. Training parameters were standardized for both the original models trained on the unaugmented dataset and the models trained on the augmented dataset. This standardization allowed for a clear comparison of the impact of data augmentation on model performance. Figure 6 offers visual insights into the dataset and the impact of AutoAugment.

## 3. Results and Discussion

### 3.1. Evaluation of Deep Learning Models

In the comparative analysis of deep learning models for classifying pregnant sows using low-frequency ultrasound images, we employed several critical metrics to evaluate performance: Accuracy, Specificity, Precision, Recall, F1-Score, and the area under the curve (AUC). Accuracy reflects the overall correctness of predictions, while Specificity measures the model’s ability to identify nonpregnant sows (true negatives) correctly. Precision quantifies the proportion of correctly predicted positive cases (pregnant sows) out of all predicted positives, highlighting the reliability of positive predictions. Recall measures the proportion of actual positives (pregnant sows) that are correctly identified, ensuring the model’s sensitivity to positive cases. F1-Score, the harmonic mean of Precision and Recall, balances these two metrics, especially in scenarios with class imbalance. AUC quantifies the model’s capacity to distinguish between pregnant and nonpregnant classes across various decision thresholds.

The Results section presents these metrics for each model, including stratified evaluations by gestational age groups (15–17 days, 18–21 days, and 22–25 days). This stratification provides deeper insights into model performance at different pregnancy stages. A comprehensive table summarizing all metrics and their corresponding values is included to ensure consistency with the Results section.

### 3.2. Quantitative Performance Metrics

Accuracy is the primary measure of model effectiveness, indicating the proportion of total predictions correctly identified. Following data augmentation, our study noted a significant improvement in accuracy rates across the evaluated models, rising from an initial average of 78.20% to 80.51%. This improvement highlights the importance of data augmentation in overcoming the challenges of imbalanced datasets and the intricate features of low-frequency ultrasound images in diagnostics.

Our models have shown a notable increase in specificity, from 81.82% to 82.91%. This enhancement in the models’ ability to correctly identify nonpregnant cases significantly reduces the risk of false positive diagnoses, thereby avoiding unnecessary interventions.

The F1-Score, a metric balancing precision and recall, improved from 72.28% to 75.59%, reflecting the models’ improved performance in managing the trade-off between identifying all relevant instances and minimizing false positives. The AUC, representing the model’s capability to differentiate between classes across various thresholds, increased from 0.834 to 0.862, suggesting an overall enhancement in model reliability and diagnostic confidence.

Considering potential class imbalances in the datasets—a frequent issue in agricultural data where one outcome may significantly exceed another—relying solely on Accuracy and Specificity might bias performance evaluation toward the majority class. To mitigate this, the F1-Score and AUC were incorporated as complementary metrics [34,35], providing a more nuanced and comprehensive assessment of model performance.

### 3.3. Gestational Age-Specific Insights

A comparison of the analysis results of baseline data (Table 2) and the results following AutoAugment data augmentation (Table 3) offers a detailed understanding of the impact of gestational age on model performance. Initially, at the ‘early gestation’ stage (15–17 days), baseline assessments of the models demonstrate moderate diagnostic efficiency. However, there is a significant improvement post-augmentation, as evidenced by increased F1-Scores and accuracy rates. This highlights the augmented models’ enhanced ability to detect pregnancy in its early stages.

During ‘mid gestation’ (18–21 days), the baseline models already exhibit a higher accuracy level compared to the ‘early gestation’ phase, reflecting the relative ease of detecting pregnancy as gestational signs become more evident. Post-augmentation, there is further improvement in performance, particularly with the Transformer-based ViT-H model. This model shows a notable rise in accuracy and F1-Score, emphasizing its effectiveness in mid-stage pregnancy diagnostics.

The baseline models perform well for ‘late gestation’ (22–25 days), attributed to the distinct gestational indicators at this stage. However, the improvements observed post-augmentation, especially regarding AUC values for the ConvNeXt-xlarge model, are significant. This suggests a considerable enhancement in diagnostic precision, confirming the model’s robustness in late-stage pregnancy diagnosis.

This comparative analysis highlights the varying impacts of different gestational stages on model performance. It demonstrates the substantial benefits data augmentation offers across all gestational periods. Notably, the augmented models show marked improvements in diagnostic capabilities at various gestational ages, reinforcing the vital role of advanced data processing techniques in boosting the effectiveness of ultrasound-based pregnancy detection.

### 3.4. Comprehensive Discussion

Comprehensive analysis across different gestational periods clearly revealed the significant impact of data augmentation on the performance of deep learning models. Although the Xception model experienced a slight drop in specificity, it demonstrated itself to be an effective tool for early pregnancy detection. This effectiveness is attributed to its advanced feature extraction capabilities. Conversely, the ViT-H and ConvNeXt-xlarge models demonstrated their respective strengths during the mid and late gestation periods. This study’s most notable outcome is the ability of the deep learning models to detect pregnancy as early as 15–17 days. At this stage, physiological indicators such as uterine echogenicity changes are minimal and highly challenging for human experts to distinguish. Our deep learning models achieved substantial accuracy early on, showcasing their advanced feature recognition capabilities and surpassing traditional methods and human expertise in this context.

Table 2 and Table 3, which detail performance metrics and comparisons, further emphasize the valuable insights gained from this study. They illustrate the nuanced differences in model performances at various gestational stages, providing a clear picture of each model’s strengths and weaknesses.

The empirical evidence gathered in this research suggests that strategic implementation of data augmentation can revolutionize pig pregnancy diagnostics. This advancement has the potential to greatly enhance the accuracy and efficiency of pig pregnancy diagnostics, offering a more precise, efficient, and non-invasive approach to managing sow pregnancies. This development not only contributes to the field of veterinary diagnostics but also holds significant implications for improving operational efficiency in the pig industry. Furthermore, the improvement in AUC values after data augmentation highlights the capability of deep learning models to process subtle signal variations in low-frequency ultrasound data, previously considered a limitation in practical pig pregnancy diagnosis.

By enhancing accuracy across different pregnancy stages, these advancements not only ensure precise and timely diagnoses but also reduce the risk of misdiagnosis, which could lead to unnecessary interventions or delays in providing care for pregnant sows. Particularly at 15–17 days, when traditional methods often fall short, this deep learning-based approach offers a groundbreaking solution, emphasizing its practical value and potential to transform swine production management.

## 4. Conclusions

This research marks a significant advancement in applying low-frequency ultrasound imaging for precise sow pregnancy diagnosis, a crucial aspect of managing and sustaining the economic sustainability of livestock farms. The study’s methodical approach encompassed data collection, expert classification, and rigorous filtering, enabling the segmentation of data into distinct training and testing datasets. By employing advanced deep learning models—specifically, the transformer-based ViT-H and the CNN-based ConvNeXt-xlarge and Xception models—this research demonstrated a considerable improvement in the accuracy of diagnosing sow pregnancies using low-frequency ultrasound images. The implementation of AutoAugment’s Cifar-10 policy for data augmentation significantly enhanced these models’ performance, highlighting the transformative potential of machine learning technologies in modernizing agricultural practices.

The study’s findings lay a solid foundation for future research, addressing existing limitations and broadening the application scope. One notable limitation is using a single low-frequency ultrasound device, suggesting the need for incorporating a more comprehensive range of devices to reflect the diversity encountered in real-world agricultural settings more accurately. Moreover, the variability in image capturing by different experts indicates the necessity for a more comprehensive data collection strategy. This strategy should include various devices and operational contexts from multiple livestock farms, potentially enhancing the robustness and general applicability of the models. Expanding the data source to include information directly from farmworkers for model training could further improve the models’ performance and reliability in practical field conditions.

In summary, the findings of this study represent a significant stride toward integrating deep learning technologies in the agricultural sector, particularly in livestock management. This research not only contributes to improving farm operational efficiency but also aligns with the broader goal of leveraging technological advancements to meet the growing demands of contemporary society. By offering a reliable, efficient, and scalable solution for sow pregnancy diagnosis, this study has the potential to significantly impact the agricultural industry, paving the way for more innovative and technologically advanced farming practices.

Additionally, the broader implications of these findings extend to cost reduction, operational efficiency, and animal welfare within the swine industry. The automation and precision achieved through deep learning models can streamline diagnostic processes, reduce resource waste, and optimize breeding schedules. By minimizing unnecessary interventions, the proposed solution supports sustainable farming practices that enhance productivity while promoting animal welfare. These advancements highlight the transformative potential of technological integration in modern livestock management.

## Figures and Tables

**Table 1:** Types and number of ultrasound image data.

Dataset	Days	Train	Validation	Test
Original Dataset	15–17	5613	1403	1696
	18–21	9309	2327	2898
	22–25	7829	1957	2430
Augment Dataset	15–17	140,320	35,080	1696
	18–21	232,720	58,180	2898
	22–25	195,720	48,930	2430

**Table 2:** Overall performance of the original data (Unit: %).

Model	Days	Accuracy (%)	Specificity (%)	Precision (%)	Recall (%)	F1-Score (%)	AUC
ViT-H	15–17	64.564	77.249	43.441	37.407	40.199	0.644
	18–21	78.778	76.644	83.803	80.195	81.960	0.858
	22–25	87.942	84.083	86.360	91.444	88.830	0.955
	Average	77.095	79.325	71.201	69.682	70.329	0.819
ConvNeXt xlarge	15–17	70.401	78.114	53.493	53.889	53.690	0.732
	18–21	77.536	81.228	85.770	75.086	80.073	0.858
	22–25	85.556	88.149	88.555	83.203	85.795	0.936
	Average	77.831	82.497	75.939	70.726	73.186	0.842
Xception	15–17	68.632	80.709	50.881	42.778	46.479	0.681
	18–21	81.850	80.709	86.582	82.606	84.548	0.890
	22–25	88.560	89.533	90.226	87.677	88.933	0.955
	Average	79.680	83.651	75.897	71.020	73.320	0.842
Composite Average of Model’s Results	15–17	67.866	78.691	49.272	44.691	46.789	0.686
	18–21	79.388	79.527	85.385	79.296	82.194	0.868
	22–25	87.353	87.255	88.380	87.441	87.853	0.949
	Average	78.202	81.824	74.346	70.476	72.278	0.834

**Table 3:** Overall performance of the proposed method (Unit: %).

Model	Days	Accuracy (%)	Specificity (%)	Precision (%)	Recall (%)	F1-Score (%)	AUC
ViT-H	15–17	72.995	83.997	59.071	49.444	53.831	0.756
	18–21	80.745	75.433	83.790	84.271	84.030	0.892
	22–25	87.778	88.754	89.491	86.892	88.172	0.947
	Average	80.506	82.728	77.450	73.536	75.344	0.865
ConvNeXt xlarge	15–17	71.757	82.180	56.448	49.444	52.715	0.739
	18–21	81.988	77.682	85.138	84.845	84.991	0.886
	22–25	86.049	89.619	89.787	82.810	86.158	0.943
	Average	79.931	83.160	77.125	72.366	74.621	0.856
Xception	15–17	73.644	81.920	59.100	55.926	57.469	0.764
	18–21	82.678	80.363	86.600	84.214	85.390	0.894
	22–25	86.914	86.246	87.520	87.520	87.520	0.942
	Average	81.078	82.843	77.740	75.886	76.793	0.866
Composite Average of Model’s Results	15–17	72.799	82.699	58.206	51.605	54.671	0.753
	18–21	81.804	77.826	85.176	84.443	84.804	0.891
	22–25	86.914	88.206	88.933	85.740	87.283	0.944
	Average	80.505	82.910	77.438	73.930	75.586	0.862

## Data Availability

Upon reasonable request, the datasets of this study can be available from the corresponding author.
